# Cardiac safety of trabectedin monotherapy or in combination with pegylated liposomal doxorubicin in patients with sarcomas and ovarian cancer

**DOI:** 10.1002/cam4.3903

**Published:** 2021-05-07

**Authors:** Robin L. Jones, Thomas J. Herzog, Shreyaskumar R. Patel, Margaret von Mehren, Scott M. Schuetze, Brian A. Van Tine, Robert L. Coleman, Roland Knoblauch, Spyros Triantos, Peter Hu, Waleed Shalaby, Tracy McGowan, Bradley J. Monk, George D. Demetri

**Affiliations:** ^1^ Sarcoma Unit Royal Marsden Hospital/Institute of Cancer Research London UK; ^2^ University of Cincinnati Cancer Center University of Cincinnati Cincinnati OH USA; ^3^ Department of Sarcoma Medical Oncology University of Texas MD Anderson Cancer Center Houston TX USA; ^4^ Fox Chase Cancer Center Philadelphia PA USA; ^5^ Department of Internal Medicine University of Michigan Ann Arbor MI USA; ^6^ Washington University in St. Louis St. Louis MO USA; ^7^ US Oncology Research The Woodlands TX USA; ^8^ Janssen Research & Development, LLC Raritan NJ USA; ^9^ Medical Group Oncology Janssen Scientific Affairs, LLC Horsham PA USA; ^10^ Arizona Oncology (US Oncology Network) University of Arizona College of Medicine, and Creighton University School of Medicine at St. Joseph's Hospital and Medical Center Phoenix AZ USA; ^11^ Sarcoma Center Department of Medical Oncology Dana‐Farber Cancer Institute (DFCI) Harvard Medical School and Ludwig Center at Harvard Boston MA USA

**Keywords:** anthracycline, cardiac toxicity, chemotherapy, patient outcomes, soft tissue sarcomas

## Abstract

**Background:**

As with other alkylating agents, cardiac dysfunction can occur with trabectedin therapy for advanced soft tissue sarcomas (STS) or recurrent ovarian cancer (ROC) where treatment options for advanced disease are still limited. Cardiac safety for trabectedin monotherapy (T) for STS or in combination with pegylated liposomal doxorubicin (T+PLD) for ROC was evaluated in this retrospective postmarketing regulatory commitment.

**Methods:**

Patient data for multiple cardiac‐related treatment‐emergent adverse events (cTEAEs) were evaluated in pooled analyses of ten phase 2 trials, one phase 3 trial in STS (*n* = 982), and two phase 3 trials in ROC (*n* = 1231).

**Results:**

Multivariate analyses on pooled trabectedin data revealed that cardiovascular medical history (risk ratio [RR (95% CI)]: 1.90 [1.24‐2.91]; *p* = 0.003) and age ≥65 years (RR [95% CI]: 1.78 [1.12‐2.83]; *p* = 0.014) were associated with increased risk for cTEAEs. Multivariate analyses showed increased risk of experiencing cTEAEs with T+PLD compared to PLD monotherapy (RR [95% CI]: 2.70 [1.75‐4.17]; *p* < 0.0001) and with history of prior cardiac medication (RR [95% CI]: 1.88 [1.16‐3.05]; *p* = 0.010).

**Conclusions:**

For patients with STS or ROC who still have limited treatment options, trabectedin may be initiated after carefully considering benefit versus risk.

**Trial Registration** (ClinicalTrials.gov): NCT01343277; NCT00113607; NCT01846611.

## Graphical abstract

Cardiac safety for trabectedin monotherapy advanced soft tissue sarcomas (STS) or in combination with pegylated liposomal doxorubicin (T+PLD) for recurrent ovarian cancer (ROC) was evaluated in pooled analyses of 10 phase 2 trials, one phase 3 trial in STS (*n* = 982), and two phase 3 trials in ROC (*n* = 1231). Cardiovascular medical history, age ≥65 years, and prior use of anthracyclines were associated with increased risk for cTEAEs. For patients with STS or ROC who still have limited treatment options, trabectedin may be initiated after carefully considering benefit versus risk.

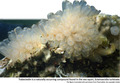

## 1 INTRODUCTION

Trabectedin is a DNA‐binding agent with a unique antitumor mechanism of action (MOA) targeting the transcription‐coupled nucleotide excision repair (NER) system. Trabectedin was developed for the treatment of soft tissue sarcomas (STS) and epithelial ovarian cancer based on its novel cytotoxic activity. These cancers still have limited treatment options, particularly where advanced disease has progressed with other therapies.^1^

Preclinical studies with trabectedin showed no toxicity in cultured rat myocytes *in vitro*, while single and repeated doses in Cynomolgus monkeys did not induce any relevant cardiac, vascular, or respiratory effects.^2^ Further, a low incidence of cardiac‐related treatment‐emergent adverse events (cTEAE) was reported in previous analyses from earlier phase 1–2 clinical trials and one phase 3 (OVA‐301), pharmacovigilance databases, and spontaneously reported cases; tachycardia or palpitations were the most common cTEAEs reported.^2^ No clinically relevant left ventricular ejection fraction (LVEF) changes occurred in phase 1 combination trials, while LVEF decreases from baseline were similar [9% of patients (pegylated liposomal doxorubicin [PLD]) and 7% (trabectedin+PLD)] with no relevant symptoms in one phase 3 trial.^2^

Trabectedin is now approved for STS in 80 countries and for ovarian cancer in combination with PLD in 71 countries. European Union (EU) approval for trabectedin+PLD for relapsed platinum‐sensitive ovarian cancer was granted in October 2009. In the United States (US), trabectedin was approved for STS treatment following the failure of anthracycline‐based chemotherapy in October 2015. Approval in the United States was contingent upon undertaking post‐marketing requirements to characterize risk of cardiotoxicity and its sequelae with trabectedin to identify risk factors including previous treatments known to be cardiotoxic (e.g., anthracyclines).

As an extension to the cTEAE analysis reported in 2011^2^, we now report the findings of this retrospective pooled analysis of key cTEAEs for all patients enrolled in ten phase 2 trials and one phase 3 trial involving trabectedin monotherapy (T) for STS and other solid tumors and two phase 3 trials in combination with PLD for recurrent ovarian cancer (ROC).

## 2 METHODS

### 2.1 Overall safety evaluation plan and description of safety studies

Safety analysis sets incorporated two pooled analyses: Cardiac safety with T was evaluated using data from ten phase 2 and one phase 3 trial (SAR‐3007 [NCT01343277]) in STS and other solid tumors at a dose and regimen of 1.5 mg/m^2^ every 3 weeks (q3wk), 24 h. Cardiac safety with combination trabectedin+PLD was derived from two phase 3 ovarian cancer trials (OVA‐301 [NCT00113607] and OVC‐3006 [NCT01846611]) where trabectedin (1.1 mg/m^2^ q3wk; 3 h) was co‐administered with PLD (30 mg/m^2^ q3wk; 90 min). Phase 3 trial designs are described in Table 1. Key inclusion/exclusion criteria for enrollment are presented for each phase 3 trial (Table S1), cardiac safety evaluations by individual trial (Table S2), and exposure and cancer diagnoses for pooled data from phase 2 and 3 studies (Table S3). Study protocols and amendments were reviewed by an Independent Ethics Committee or an Institutional Review Board.

**TABLE 1:** Overview of study designs for phase 3 trials

Protocol	Study description	Treatment groups	Patients in safety analysis set, n
Phase 3 soft tissue sarcoma study – single‐agent therapy			
SAR‐3007 (NCT01343277)	A multicenter, open‐label, randomized, active‐controlled, parallel‐group phase 3 study comparing the safety and efficacy of trabectedin versus dacarbazine among adults with unresectable, locally advanced or metastatic L‐sarcoma, previously treated with at least an anthracycline and ifosfamide‐containing regimen or an anthracycline‐containing regimen and one additional cytotoxic chemotherapy regimen. A normal LVEF at baseline was not required for enrollment.	Trabectedin Arm: 1.5 mg/m^2^ as a 24 h IV infusion q3wk.	378
		Dacarbazine Arm: 1 g/m^2^ as a 20‐ to 120‐min IV infusion q3wk.	172
Phase 3 ovarian cancer studies – combination therapy			
OVA‐301 (NCT00113607)	A multicenter, open‐label, randomized study to assess the safety and efficacy of trabectedin+PLD versus PLD in patients with ROC treated with only one platinum‐based chemotherapy regimen. Patients with a normal LVEF at baseline were eligible to enroll in the study.	Trabectedin+PLD Arm: PLD, 30 mg/m^2^ as a 90‐min infusion immediately followed by a 3 h trabectedin IV infusion 1.1 mg/m^2^ q3wk.	333
		PLD Arm: PLD, 50 mg/m^2^ as a 90‐min infusion q4wk.	330
OVC‐3006 (NCT01846611)	A multicenter, open‐label, randomized study to assess the efficacy and safety of trabectedin+PLD as third line chemotherapy in patients with platinum‐sensitive ROC who received two previous lines of platinum‐based chemotherapy. Patients with a normal LVEF at baseline were eligible to enroll in the study.	Treatment Arm A: PLD 30 mg/m^2^ as a 90‐min infusion immediately followed by a 3 h trabectedin infusion 1.1 mg/m^2^ q3wk.	286
		Treatment Arm B: PLD, 50 mg/m^2^ as a 90‐min infusion q4wk.	282

Abbreviations: IV, intravenous; L‐sarcoma, leiomyosarcoma or liposarcoma; LVEF, left ventricular ejection fraction; PLD, pegylated liposomal doxorubicin; q3wk, once every 3 weeks; q4wk, once every 4 weeks; ROC, recurrent ovarian cancer.

### 2.2 Statistical methodology

#### 2.2.1 Definition of subgroups and general analysis methods

Continuous variables were summarized using descriptive statistics (i.e., mean, standard deviation [SD], median, and range) and categorical variables by frequency counts and percentages. Time‐to‐event variable data were summarized by Kaplan‐Meier methods for 25th, 50th, and 75th percentiles with 95% confidence intervals (CIs). Treatment group comparisons are presented by hazard ratios and 95% CI from Cox proportional hazards models. Anthracycline exposure data are summarized for subjects who received anthracyclines prior to the study (i.e., prior anthracycline) and for subjects who received anthracyclines prior to and during the study (i.e., cumulative anthracycline).

Two parameters were used for the cardiac safety analysis: LVEF significant decline where available and cardiac‐related AEs of special interest (cardiac‐related AEs). Cross tabulation of ECG data were included when available.

#### 2.2.2 Cardiac‐related adverse events

cTEAEs are summarized from time of first administration to 30 days after the last dose and graded using Common Terminology Criteria for Adverse Events (CTCAE; version 4.0). Incidences of cTEAEs are defined by eight Medical Dictionary for Regulatory Activities (MedDRA) high‐level group terms (HLGTs) and associated preferred terms (PTs) and two Standardized MedDRA Queries (narrow SMQ) (Table 2). HLGTs included cardiac and vascular investigations (excluding enzyme tests), cardiac arrhythmias, cardiac disorder signs and symptoms, coronary artery disorders, endocardial disorders, heart failures, myocardial disorders, and pericardial disorders. TEAEs were coded to MedDRA version 16.0 for OVA‐301, SAR‐3007, and pooled safety analysis for T. TEAEs in OVA‐301 were aligned to MedDRA version 19.0 to match the MedDRA version of OVC‐3006 and cTEAEs presented by HLGTs and SMQs and related PTs.

**TABLE 2:** MedDRA HLGT and standardized MedDRA queries (narrow SMQ)

	cTEAEs
HLGT	Cardiac and vascular investigations (excluding enzyme tests)
	Cardiac arrhythmias
	Cardiac disorder signs and symptoms
	Coronary artery disorders
	Endocardial disorders
	Heart failure
	Myocardial disorders
	Pericardial disorders
SMQ	Cardiac failure
	Cardiomyopathy

Abbreviations: cTEAEs, cardiac‐related treatment‐emergent adverse events; HLGT, high‐level group term; MedDRA, Medical Dictionary for Regulatory Activities; SMQ, standardized MedDRA query.

#### 2.2.3 Left ventricular ejection fraction

LVEF significant decline was defined as absolute decrease ≥15%, or <lower limit of normal and absolute decrease ≥5%. LVEF recovery for subjects with significant LVEF decline was defined as either return to baseline values or <Grade 2 ejection fraction decreased toxicity (CTCAE v4.0). In all three phase 3 studies (SAR‐3007, OVA‐301, and OVC‐3006), LVEF assessments were performed at baseline and end of treatment. Additionally, OVC‐3006 was amended to provide comprehensive cardiac evaluations of patients while on treatment. Collection time points for LVEF in each study are described in Table S2.

## 3 RESULTS

### 3.1 Overall exposure

Table 3 shows the number of patients exposed to study drug by individual trial and by pooled safety analysis sets for T and trabectedin+PLD. Nine hundred and eighty‐two patients were exposed to T, and 619 patients were treated with trabectedin+PLD.

**TABLE 3:** Number of patients exposed to study treatment and safety analysis sets

Study	No. patients in safety analysis set	
Monotherapy	Trabectedin	Dacarbazine
SAR‐3007	378	172
10 phase 2 Studies	604	–
Pooled Data (SAR‐3007+phase 2 Studies)	982	–
Combination Therapy	Trabectedin+PLD	PLD
OVA‐301	333	330
OVC‐3006	286	282
Pooled Data (OVA‐301+OVC‐3006)	619	612

Abbreviation: PLD, pegylated liposomal doxorubicin.

### 3.2 Baseline characteristics: trabectedin monotherapy (T)

#### 3.2.1 Demographic characteristics

Patients treated with trabectedin (*N* = 982) had a median (range) age of 54 (12–81) years (Table S4). Most patients were female (61.6%), white (50.6%), from North America (58.2%) or Western Europe (36.7%), had an Eastern Cooperative Oncology Group performance score of 0 or 1 (99.8%), and a diagnosis of STS (88.0%). Prior anthracycline use was reported for 71.3% (Table 4). Prior anthracycline dose was only captured in the phase 3 SAR‐3007 study. A cumulative dose of prior anthracycline was reported for 337/378 subjects in the trabectedin treatment group and 162/172 subjects in the dacarbazine group. Median cumulative prior anthracycline dose was 270.00 mg/m^2^ in the trabectedin group and 240.75 mg/m^2^ in the dacarbazine group.

**TABLE 4:** Disease characteristics for patients treated with trabectedin 1.5 mg/m^2^ q3wk; 24 h (trabectedin ‐ pooled phase 2 and 3 studies)

Number (%)	Patients treated with trabectedin 1.5 mg/m^2^ q3wk; 24 h (*N* = 982)
Cancer type	
STS, L‐type	661 (67.3)
STS, Non‐L‐type	203 (20.7)
Ovarian	54 (5.5)
Breast	26 (2.6)
Renal	21 (2.1)
Melanoma	12 (1.2)
Prostate	5 (0.5)
Prior anthracycline treatment	700 (71.3)

Data are presented as No. (%).

Abbreviations: L‐type, leiomyosarcoma or liposarcoma; STS, soft tissue sarcomas; q3wk, once every 3 weeks.

#### 3.2.2 Cardiovascular medical history

Pooled analyses of T, cardiovascular medical history categorized under the vascular disorder and/or cardiac disorder system organ class (SOC) was reported for 355 (36.2%) of 982 trabectedin‐treated patients. The most commonly reported cardiovascular medical history for patients treated with trabectedin were hypertension (24.8% [244/982]), followed by deep vein thrombosis (2.9% [28/982]), hot flush (2.2% [22/982]), and coronary artery disease (2.1% [21/982]).

### 3.3 Baseline characteristics: trabectedin in combination with PLD

#### 3.3.1 Demographic characteristics

In the pooled analysis of trabectedin+PLD, patient demographic characteristics were consistent in the all‐female study populations across the trabectedin+PLD (*N* = 619) and PLD monotherapy (*N* = 612) groups, with median (range) ages of 58 (26–83) and 59 (27–91) years, respectively. Most patients were white (84% and 82%, respectively) (Table S5), and baseline disease characteristics were consistent across both treatment groups (Table 5).

**TABLE 5:** Disease characteristics at baseline for treated patients (pooled studies ET743‐OVC‐3006 and ET743‐OVA‐301 studies)

Number (%)	Trabectedin+PLD (*N* = 619)	PLD (*N* = 612)
Histology		
Papillary/Serous	412 (66.6)	420 (68.6)
Other	97 (15.7)	92 (15.0)
Endometrioid	38 (6.1)	37 (6.0)
Clear Cell Carcinoma	24 (3.9)	21 (3.4)
Peritoneal Carcinoma	21 (3.4)	17 (2.8)
Fallopian Tube Carcinoma	10 (1.6)	15 (2.5)
Mixed Epithelial Tumor	7 (1.1)	5 (0.8)
Mucinous (exclusion)	5 (0.8)	3 (0.5)
Transitional Carcinoma (Brenner)	5 (0.8)	2 (0.3)
Prior anthracycline treatment	38 (6.1)	36 (5.9)
Time from initial diagnosis to randomization, median (range), months	24.25 (6.6, 169.3)	25.17 (2.5, 230.4)

Data are presented as No. (%) unless otherwise specified.

Abbreviation: PLD, pegylated liposomal doxorubicin.

#### 3.3.2 Cardiovascular medical history

In pooled analysis of trabectedin+PLD, 45.6% and 51.5% of patients in the trabectedin+PLD and PLD monotherapy groups, respectively, had prior cardiovascular medical history reported for vascular and/or cardiac disorders SOC. These included: hypertension (30.5% [189/619] in the trabectedin+PLD group and 34.6% [212/612] in the PLD monotherapy group), followed by myocardial ischemia (6.0% [37/619] and 6.7% [41/612], respectively), varicose vein (3.9% [24/619] and 3.8% [23/612], respectively), deep vein thrombosis (2.7% [17/619] and 2.5% [15/612], respectively), and hot flush (1.8% [11/619] and 2.9% [18/612], respectively).

### 3.4 Cardiac safety results

#### 3.4.1 Trabectedin monotherapy (T)

In the pooled analysis of T, 110 (11.2%) patients who received ≥1 trabectedin dose experienced a cTEAE (Table S6). cTEAEs reported for ≥1% of trabectedin‐treated patients included tachycardia (3.1%), palpitations (1.5%), LVEF decrease (1.3%), sinus tachycardia (1.0%), and congestive cardiac failure (1.0%). Median time from the first dose of study drug to the onset of first occurrence of a cTEAE for trabectedin‐treated patients was 40 days. For 65% of patients with cTEAE, the event was reported as resolved, with a median time to resolution of 8 days. Thirty‐seven (3.8%) trabectedin‐treated patients experienced a Grade 3 or 4 cTEAE (Table S7). Cardiac‐related serious TEAEs (SAEs) were reported in 36 (3.7%) trabectedin‐treated patients (Table S8); those most frequently reported (≥5 patients) included: congestive cardiac failure (0.8%), pulmonary edema (0.6%), ejection fraction decreased (0.5%), cardiac failure (0.5%), and atrial fibrillation (0.5%). Six (0.6%) trabectedin‐treated patients experienced a cTEAE leading to death (Table S9).

#### 3.4.2 Unique study features

In the phase 3 study comparing trabectedin versus dacarbazine (SAR‐3007), cTEAEs were reported for 58 (15.3%) trabectedin patients and 25 (14.5%) dacarbazine patients; however, cardiac failure (5.0% vs. 2.3%), cardiomyopathy (3.7% vs. 2.3%), and heart failure (2.9% vs. 0.6%) were higher with trabectedin. Furthermore, the median cumulative prior anthracycline dose was greater in the trabectedin group (329.75 mg/m^2^) compared to the dacarbazine group (180.00 mg/m^2^), which should also be taken into consideration. Among patients with a cTEAE, more patients in the trabectedin group (39 [67.2%] of 58) received a prior cumulative anthracycline dose ≥300 mg/m^2^ compared with the dacarbazine group (10 [40.0%] of 25). Median time from the first dose of study drug to the onset of first occurrence of a cTEAE was twice as long in the trabectedin group compared with the dacarbazine group (46 days vs. 23 days); however, the median time to resolution was twice as long in the trabectedin group (8 days vs. 4 days). In terms of prior anthracycline exposure among patients with a significant decrease in LVEF from baseline and for whom dose information was reported, prior cumulative anthracycline dose of ≥300 mg/m^2^ was reported in 22/34 (64.7%) in the trabectedin group compared with 7/11 (63.6%) patients in the dacarbazine group.

#### 3.4.3 Trabectedin in combination with PLD

cTEAEs were reported for 78 (12.6%) patients in the trabectedin+PLD group and 34 (5.6%) patients in the PLD monotherapy group; most commonly reported cTEAE was LVEF decrease (7.8% vs. 4.2%, respectively). Within these SMQ/HLGTs, palpitation was the only cTEAE reported with at least a 2% greater incidence in the trabectedin+PLD group compared with the PLD monotherapy group (3.2% vs. 1.0%) (Table S10).

**FIGURE 1:**
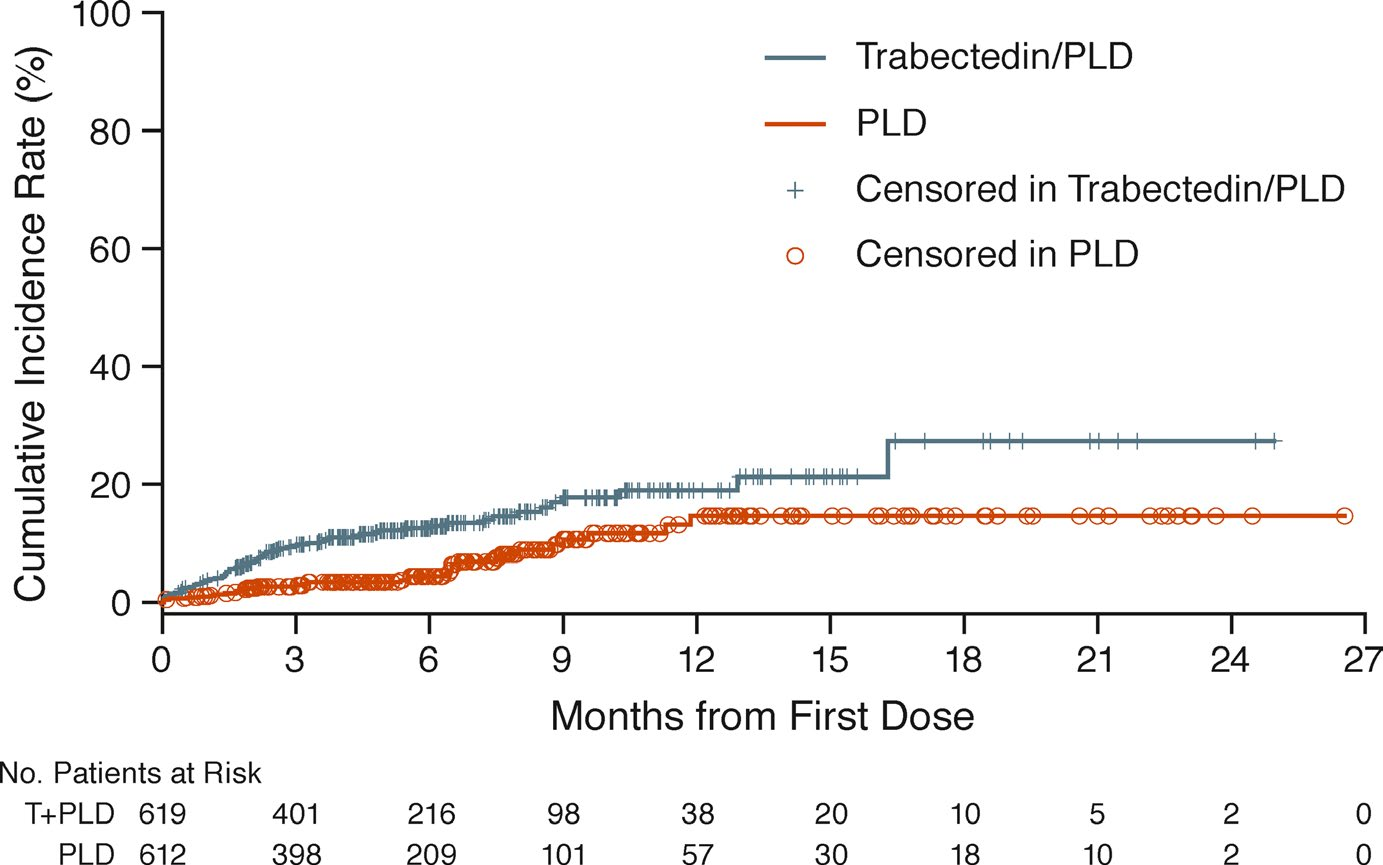
Cumulative Incidence of Cardiac‐Related Adverse Events Over Treatment Duration for Treated Patients (Pooled Studies ET743‐OVC‐3006 and ET743‐OVA‐301). PLD, pegylated liposomal doxorubicin

Kaplan‐Meier analyses showed an increased risk of cTEAEs with trabectedin+PLD compared with PLD monotherapy (Figure 1). Cumulative incident rate curves separated early and remained separated throughout treatment. Median time from first study dose to the onset of first occurrence of cTEAE was shorter with trabectedin+PLD (57 days) compared with PLD monotherapy (98 days), while most patients in both groups had similar resolutions of cTEAEs (57.1% and 55.9%) and time to resolution (8 days). However, while Grade 3 or 4 cardiac‐related events were reported more frequently with trabectedin+PLD versus PLD monotherapy (14 [2.3%] vs. 4 [0.7%] patients; Table S11); no cTEAEs were reported with an incidence of ≥1% in either group. Last, cardiac‐related SAEs were reported more frequently with trabectedin+PLD (11 [1.8%]) vs. PLD monotherapy (3 [0.5%]) (Table S12). Congestive heart failure was quite low in both the combination (3 [0.5%]) and monotherapy groups (1 [0.2%]).

#### 3.4.4 Unique study features

In OVA‐301, fewer patients with a significant decrease from baseline in LVEF in the trabectedin+PLD group had a cardiovascular medical history compared with the PLD monotherapy group (23.8% vs. 52.6%). In OVC‐3006, median cumulative PLD dose for patients with a cTEAE was lower with trabectedin+PLD treatment compared with PLD monotherapy (180.78 vs. 329.67 mg/m^2^). Median cumulative anthracycline dose of ≥300 mg/m^2^ was reported in 11/43 (25.6%) in trabectedin+PLD patients with a cTEAE compared to 17/23 (73.9%) PLD monotherapy patients.

In OVC‐3006, the median time from first dose of drug to the onset of first occurrence of a cTEAE was shorter with trabectedin+PLD compared with PLD monotherapy (68 days vs. 169 days); however, the median time to resolution was longer with PLD monotherapy group compared with trabectedin+PLD (29 days vs. 16 days). Median cumulative PLD dose for patients having significant decreases from baseline in LVEF was lower in the trabectedin+PLD group compared with the PLD monotherapy group (149.39 vs. 251.25 mg/m^2^). In addition, median cumulative anthracycline doses of ≥300 mg/m^2^ were associated with a significant decrease from baseline in LVEF; this was reported in 3/19 (15.8%) trabectedin+PLD and 5/10 (50.0%) PLD monotherapy patients.

### 3.5 Multivariate analyses

#### 3.5.1 Trabectedin monotherapy

**FIGURE 2:**
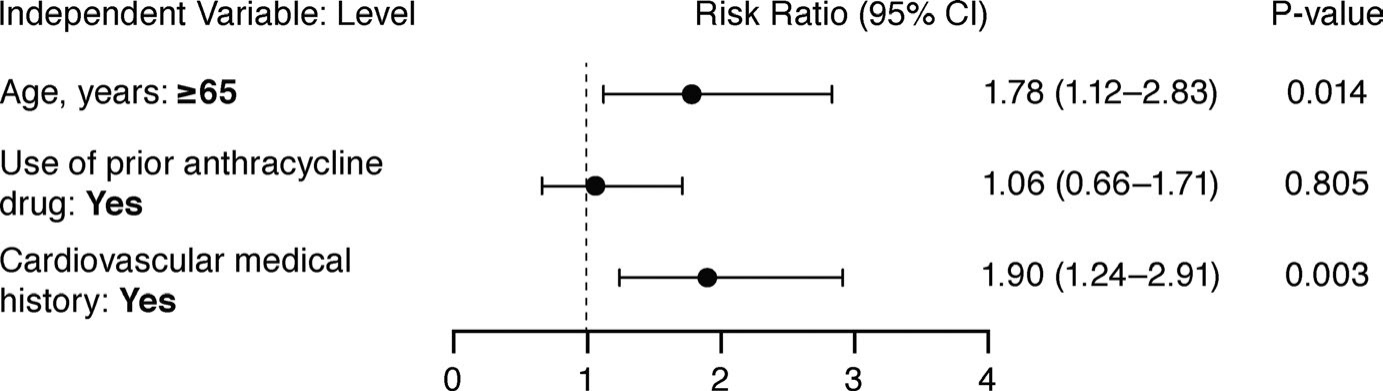
Multivariate Analysis on Incidence of Cardiac‐Related TEAEs (Logistic Regression) for Treated Patients (Trabectedin – Pooled Phase 2 and 3 Studies). Dependent variable: incidence of cardiac‐related TEAEs. CI, confidence interval; TEAEs, treatment‐emergent adverse events

Trabectedin‐treated patients who experienced a cTEAE were generally older (18.4% aged ≥65 years vs. 9.6% aged <65 years). Results from multivariate analyses of cTEAEs when controlling for potential risk factors are presented in Figure 2. These showed that patients aged ≥65 years and those with cardiovascular medical history had an increased risk of cTEAEs. The effect of cumulative anthracycline dose of ≥300 versus <300 mg/m^2^ and baseline LVEF <lower limit of normal (LLN) versus ≥LLN, however, could not be evaluated in the ten phase 2 studies due to differences in study designs.

#### 3.5.2 Trabectedin in combination with PLD

**FIGURE 3:**
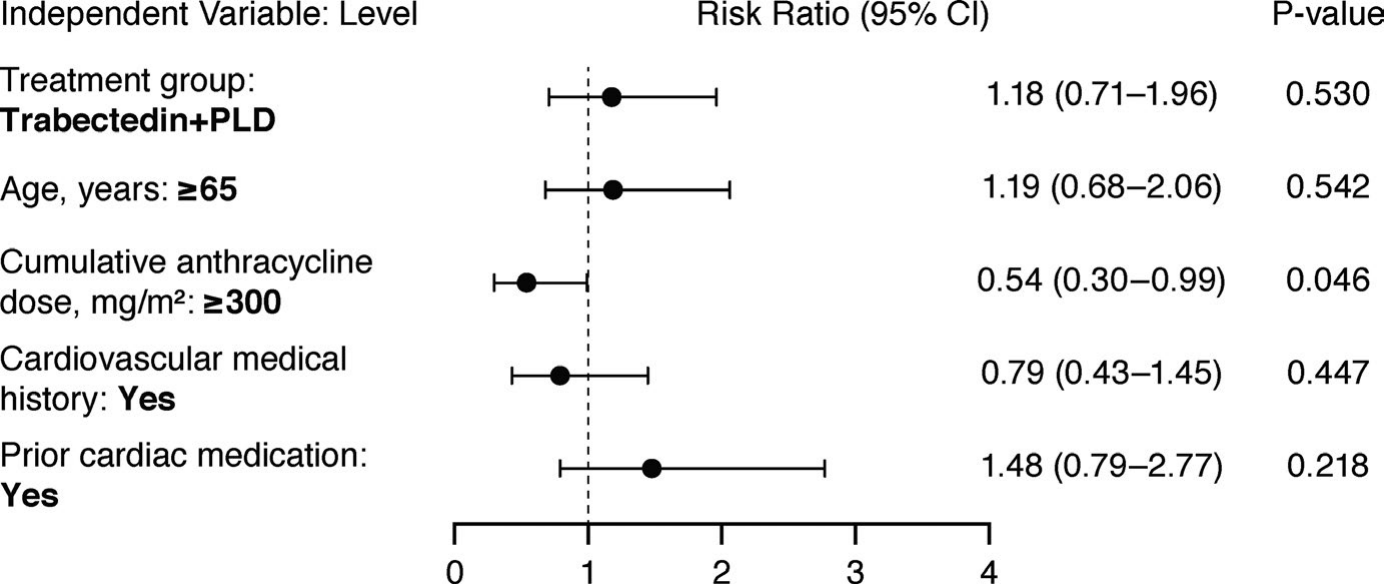
Multivariate Analysis on Incidence of Significant LVEF Decrease (Logistic Regression) for Treated Patients (Pooled Studies OVC‐3006 and OVA‐301). Dependent variable: incidence of significant LVEF decrease. CI, confidence interval; LVEF, left ventricular ejection fraction; PLD, pegylated liposomal doxorubicin

In the multivariate analyses, when controlling for potential risk factors, results showed that patients receiving trabectedin+PLD were at increased risk for experiencing a cTEAE compared with PLD monotherapy (risk ratio [RR] 2.70; 95% CI: 1.75–4.17; *p* < 0.0001). Furthermore, patients with a history of prior cardiac medication use who received trabectedin+PLD versus PLD were also at increased risk of experiencing cTEAEs (RR 1.88; 95% CI: 1.16–3.05; *p* = 0.010). Patients with a cumulative anthracycline dose of ≥300 mg/m^2^ who received trabectedin+PLD in the OVC‐3006 and OVA‐301 trials were at increased risk for a significant decrease in LVEF compared with patients who received PLD monotherapy (RR 0.54; 95% CI: 0.30–0.99; *p* = 0.046) (Figure 3).

## 4 DISCUSSION

Trabectedin was developed based on its novel chemical structure and promising preclinical activity in several types of human tumors. The development program focused on STS^3^ and ROC^4^, ^5^ in which trabectedin was active at very low concentrations in both preclinical models and clinical trials.^1^ Trabectedin binds to the N2 position of guanine in the minor groove of DNA and bends the helix toward the major groove, a unique property in the class of DNA‐binding agents; it triggers a cascade of events affecting several transcription factors, DNA‐binding proteins, and DNA‐repair pathways (e.g., transcription‐coupled NER), resulting in slowed progression through S and G2/M phases and p53‐independent apoptosis. Trabectedin also prevents the binding of translocation‐related oncogenic fusion proteins to DNA promoter regions, thereby interfering with the function of proteins that contribute to the malignant phenotype and tumor progression.^6^, ^7^, ^8^, ^9^

PLD is doxorubicin hydrochloride encapsulated in STEALTH^®^ liposomes for intravenous administration. PLD was granted approvals for advanced ovarian cancer in June 1999 and October 2000 in the United States and European Union, respectively. As with any anthracycline, PLD can cause myocardial damage, including congestive heart failure, as the total cumulative dose of doxorubicin hydrochloride approaches 550 mg/m^2^. In a clinical study of 250 patients with advanced cancer who were treated with PLD, the risk of cardiotoxicity was 11% when the cumulative anthracycline dose was 450 to 550 mg/m^2^.^10^

This is the most comprehensive analysis of cardiac safety in the setting of trabectedin administration from clinical trial data including more than 1600 patients. Strengths include pooled analyses of one phase 3 trial and ten phase 2 trials of T in STS and two phase 3 trials of trabectedin in combination with PLD for ROC. Limitations include patient heterogeneity and varying dosing, scheduling, and infusion times for T (1.5 mg/m^2^ q3wk; 24 h) compared with PLD combination therapy (trabectedin 1.1 mg/m^2^ q3wk; 3 h). The authors recognize that cardiac adverse events with diverse etiologies make it difficult to ascribe the outcomes to trabectedin alone or identify specific causal mechanisms. Cardiotoxicity may be mediated by multiple mechanisms including damage from prior cardiotoxic therapies (anthracyclines), preexisting cardiovascular comorbidities, and the alkylating MOA among others. Last, the retrospective nature of data collection and other events, such as sepsis (that could contribute to cardiac events) are additional limitations.

In phase 3 SAR‐3007 study^3^, no difference in the overall incidence rate of any‐grade cTEAEs was observed between trabectedin‐ and dacarbazine‐treated patients. Multivariate analysis of safety data (data not shown) indicated that cumulative anthracycline dose of ≥300 mg/m^2^ and baseline LVEF <LLN were risk factors for the development of cTEAEs in STS. In pooled analyses, only age ≥65 years and cardiovascular medical history were associated with an increased risk of cTEAEs. This difference could be attributed to variability in patient populations, pretreatment history, and consistent baseline LVEF testing in the ten phase 2 studies compared with SAR‐3007. In summation, cardiac safety signals observed with T may be, in part, due to the history of prior or concurrent therapy with an anthracycline, known for potential short‐ and long‐term cardiotoxicity, and longer median duration of treatment for patients receiving trabectedin.

In OVA‐301 and OVC‐3006, patients in the trabectedin+PLD groups experienced cTEAEs at a higher incidence, regardless of toxicity grade, compared with PLD monotherapy patients. In OVA‐301, multivariate analyses indicated an increased risk of cTEAEs among patients in the trabectedin+PLD group compared with PLD monotherapy (data not shown). In OVC‐3006, however, a cumulative anthracycline dose of ≥300 mg/m^2^ and prior cardiac medication use were also identified as independently associated with increased risk of cTEAEs (data not shown). Differences between the two studies may be attributed to enrollment criteria for each study. Inclusion criteria for OVC‐3006 differed from OVA‐301 in that patients were allowed to have received two prior lines versus one line of chemotherapy for ROC, and prior PLD combination therapy was also allowed.

Ultimately, these data suggest that some patients receiving T after prior therapy with anthracyclines are at risk for cTEAEs, which may be serious in a small number. The overall risk of fatal events is relatively low but appears to be higher for patients with existing myocardial dysfunction (abnormal LVEF) or prior cardiovascular medical history. In the setting of STS, the available data support recommendations to assess LVEF by echocardiogram or multigated acquisition radionuclide scan before the initiation of trabectedin and at two‐ to three‐month intervals thereafter until trabectedin is discontinued, particularly for patients with prior cardiovascular disease. Additionally, when using trabectedin in combination with PLD for ROC, cumulative anthracycline dose ≥300 mg/m^2^ and prior cardiac medication may increase the risk of cTEAEs if prior lines of therapy involved PLD.

In conclusion, as with any systemic cytotoxic therapy, benefit versus risk should be carefully considered when instituting treatment with trabectedin in patients with few other treatment options and with risk factors for developing cTEAEs. In consultation with a cardiologist or cardio‐oncology service, baseline cardiovascular risk should be comprehensively assessed before commencing treatments with cardiotoxic potential as noted above.^11^, ^12^ Cardiotoxicity risk can be minimized by primary prevention strategies; signs and symptoms of myocardial toxicity including decreases in LVEF should be assessed routinely as described above. Dose reductions or temporary or permanent discontinuation of trabectedin should be considered when serious cTEAEs occur. Once the decision is made that benefits of trabectedin therapy outweigh risk, patients (and caregivers) should be supported throughout treatment with a personalized surveillance program to minimize cTEAE risk and promptly address when cTEAEs do occur.

## CONFLICTS OF INTEREST

Robin L. Jones: Honoraria and consulting fees from Adaptimmune, Blueprint, Clinigen, Eisai, Epizyme, Daiichi Sankyo, Deciphera, Immune Design, Johnson & Johnson, Lilly, Merck, PharmaMar, Pfizer, Tracon, UpToDate. Thomas J. Herzog: Scientific Advisory Boards for AZ, Caris, Clovis, Genentech, GSK, Johnson & Johnson, Merck. Shreyaskumar R. Patel: Clinical trial funding from Janssen, personal fees from PharmaMar (travel for the advisory board), and personal fees from M.J. Hennessey/OncLive (presentation at an educational meeting) and has acted as a paid consultant for Bayer, Daiichi Sankyo, Eli Lilly, Epizyme, Immune Design, Janssen, and Novartis Oncology; has received grants from Blueprint Medicines; and has received personal fees from CytRx and EMD Serono. Margaret von Mehren: Support to Fox Chase Cancer Center for the conduct of SAR‐3007; member of the scientific steering committee for the study; honoraria from Janssen in the past for scientific advisory board participation. Research funding from Novartis; paid consulting for Blueprint, Deciphera, Exelixis. Scott M. Schuetze: Honoraria and travel support from Janssen for participation in scientific advisory boards; research funding to the institution for support of clinical trials of trabectedin in sarcoma. Brian A. Van Tine: Basic Science Grant Funding from Pfizer, Tracon, and Merck; consulting fees from Epizyme, Lilly, CytRX, Janssen, Immune Design, Daiichi Sankyo, Bayer, Plexxikon, and Adaptimmune; speaking fees from Caris, Janssen, and Lilly; travel support from Lilly, GSK, and Adaptimmune. Robert Coleman: Grants from NIH (2P50 CA109298; P30CA016672), Gateway Foundation, and V Foundation during the conduct of the study and grants and personal fees (research support, consulting) from AstraZeneca, Clovis, Genmab, Roche/Genentech, Janssen; grants (research support) from Merck; and personal fees (consulting) from Tesaro, Medivation, Gamamab, Agenus, Regeneron, and OncoQuest outside the submitted work. Roland Knoblauch: Employee of Janssen Oncology. Spyros Triantos: Currently employed by Janssen Research and Development. Peter Hu: Employee of Janssen Research and Development. Waleed Shalaby: Employee of Janssen Scientific Affairs, LLC; stock in Johnson and Johnson, of which Janssen is a wholly owned subsidiary. Tracy McGowan: Employee of Janssen Scientific Affairs, LLC; stock in Johnson and Johnson, of which Janssen is a wholly‐owned subsidiary. Bradley Monk: Personal fees (honorarium/consultant) from Abbvie, Advaxis, Agenus, Amgen, Aravive, Asymmetric Therapeutics, Boston Biomedical, Chemo Care, ChemoID, Circulogene, Conjupro, Easai, Geistlich, Genmab/Seattle Genetics, GOG Foundation, ImmunoGen, Immunomedics, Incyte, Laekna Health Care, Mateon (formally OXiGENE), Merck, Mersana, Myriad, Nucana, OncoMed, Oncoquest, Oncosec, Perthera, Pfizer, Precision Oncology, Puma, Regeneron, Samumed, Takeda, VBL, and Vigeo; personal fees (honorarium/consultant/speaker) from AstraZeneca, Clovis, Janssen/Johnson & Johnson, Roche/Genentech, and Tesaro/GSK. George D. Demetri: Grants, personal fees, and non‐financial support from PharmaMar—including travel to a research meeting—and from Janssen; grants from AbbVie, Adaptimmune, Bayer, Daiichi‐Sankyo, Epizyme, GlaxoSmithKline, Ignyta, Loxo Oncology, Novartis, Pfizer, and Roche; personal fees from AbbVie, Adaptimmune, Bayer, Blueprint Medicines, Daiichi‐Sankyo, Caris Life Sciences, Champions Oncology, EMD‐Serono, Epizyme, G1 Therapeutics, Ignyta, Loxo Oncology, Mirati Therapeutics, Merrimack Pharmaceuticals, M.J. Hennessey/OncLive, Novartis, Pfizer, Polaris Pharmaceuticals, Roche, Sanofi, WIRB Copernicus Group, Translate BIO, ZioPharm, RELAY Therapeutics; non‐financial support from AbbVie, Daiichi‐Sankyo, Epizyme, Novartis, and Roche; travel to present at an educational meeting for M.J. Hennessey/OncLive, Novartis, and Pfizer; travel to an advisory board meeting for Bayer, Caris Life Sciences, Daiichi‐Sankyo, EMD‐Serono, Loxo Oncology, Roche, and WIRB Copernicus Group; travel to FDA meeting for Epizyme; travel to research meeting for Adaptimmune; service as a member of the Board of Directors with support for travel to board meetings for Blueprint Medicines, Merrimack Pharmaceuticals and Translate BIO; equity for Blueprint Medicines and G1 Therapeutics; equity options for Bessor Pharmaceuticals, Caris Life Sciences, Champions Oncology, Erasca Pharmaceuticals, G1 Therapeutics, Translate BIO, and RELAY Therapeutics; a patent, issued and licensed to PharmaMar, for trabectedin use for cancer (patent from PharmaMar; no funds from this and no license to Dana Farber Cancer Center or to Dr. Demetri); and patent, issued and licensed to Novartis from Dana‐Farber Cancer Institute (DFCI), for imatinib use in gastrointestinal stromal tumor, and royalties from DFCI for that patent.

## AUTHOR CONTRIBUTIONS

Robin L. Jones, Shreyaskumar Patel, Spyros Triantos, Bradley Monk, and George Demetri: conceptualization, study design, acquisition/collection of data, analysis/interpretation of data, drafted/revised manuscript content; Thomas J Herzog, Robert Coleman and Waleed Shalaby: conceptualization, study design, analysis/interpretation of data, drafted/revised manuscript content; Tracy McGowan: conceptualization, study design, drafted/revised manuscript content; Margaret von Mehren, Scott Schuetze and Brian Van Tine: acquisition/collection of data, analysis/interpretation of data, drafted/revised manuscript content; Roland Knoblauch: acquisition/collection of data, drafted/revised manuscript content; Peter Hu: analysis/interpretation of data, drafted/revised manuscript content. All authors approved the final article and agreed to be accountable for all aspects of the work.

## ETHICAL CONSIDERATION

The studies were conducted in accordance with the ethical principles for human experimentation as defined in the Declaration of Helsinki and are registered on ClinicalTrials.gov (NCT01343277; NCT00113607; NCT01846611). Study protocols and amendments were approved by the Institutional Review Board at each site. All patients provided written informed consent prior to participation in the study.

## Supporting information

Table S1‐12

## Data Availability

The data sharing policy of the study sponsor, Janssen Pharmaceutical Companies of Johnson & Johnson, is available at https://www.janssen.com/clinical‐trials/transparency. As noted on this site, requests for access to the study data can be submitted through Yale Open Data Access (YODA) Project site at http://yoda.yale.edu.
