# Supplementary material for: Cardiac safety of trabectedin monotherapy or in combination with pegylated liposomal doxorubicin in patients with sarcomas and ovarian cancer
Source: Cancer Med. 2021 May 7;10(11):3565–74. doi: 10.1002/cam4.3903 (PMC8178483; doi:10.1002/cam4.3903)
Supplement: Supplementary file 1 — Table S1‐12 [file CAM4-10-3565-s001.docx]

Online-Only Material

**Cardiac Safety of Trabectedin Monotherapy or in Combination with Pegylated Liposomal Doxorubicin in Patients with Sarcomas and Ovarian Cancer**

**CONTENTS**

**Table S1.** Key Inclusion and Exclusion Criteria for Phase 3 Trials

**Table S2**. Key Cardiac Evaluations for Phase 3 Trials

**Table S3.** Number of Patients Receiving Trabectedin Monotherapy at 1.5 mg/m^2^ q3wk; 24 h (Trabectedin - Pooled Phase 2 and 3 Studies)

**Table S4.** Demographics and Disease Characteristics for Patients Treated With Trabectedin 1.5 mg/m^2^ q3wk; 24 h (Trabectedin - Pooled Phase 2 and 3 Studies)

**Table S5.** Baseline Demographics for Treated Patients (Pooled Studies ET743-OVC-3006 and ET743-OVA-301)

**Table S6.** Cardiac-Related Treatment-Emergent Adverse Events for Patients Treated With Trabectedin 1.5 mg/m^2^ q3wk; 24 h (Trabectedin - Pooled Phase 2 and 3 Studies)

**Table S7.** Cardiac-Related Treatment-Emergent Grade 3 or 4 Adverse Events for Patients Treated With Trabectedin 1.5 mg/m^2^ q3wk; 24 h (Trabectedin - Pooled Phase 2 and 3 Studies)

**Table S8.** Cardiac-Related Serious Treatment-Emergent Adverse Events for Patients Treated With Trabectedin 1.5 mg/m^2^ q3wk; 24 h (Trabectedin - Pooled Phase 2 and 3 Studies)

**Table S9.** Cardiac-Related Treatment-Emergent Adverse Events Leading to Death for Patients Treated With Trabectedin 1.5 mg/m^2^ q3wk; 24 h (Trabectedin - Pooled Phase 2 and 3 Studies)

**Table S10.** Cardiac-Related Treatment-Emergent Adverse Events for Treated Patients (Pooled Studies ET743-OVC-3006 and ET743-OVA-301)

**Table S11.** Cardiac-Related Treatment-Emergent Grade 3 or 4 Adverse Events for Treated Patients (Pooled Study ET743-OVC-3006 and ET743-OVA-301)

**Table S12.** Cardiac-Related Serious Treatment-Emergent Adverse Events for Treated Patients (Pooled Studies ET743-OVC-3006 and ET743-OVA-301)

**Table S1.** **Key Inclusion and Exclusion Criteria for Phase 3 Trials**

| **Protocol** | **Key Inclusion Criteria** | **Key Exclusion Criteria** |
| --- | --- | --- |
| SAR-3007  (NCT01343277) | • Histologically proven, unresectable, locally advanced or metastatic liposarcoma (dedifferentiated, myxoid round cell, or pleomorphic) or leiomyosarcoma.  • Treated in any order with at least: an anthracycline and ifosfamide containing regimen; or an anthracycline containing regimen and 1 additional cytotoxic chemotherapy regimen.  • ECOG performance status score of 0 or 1.  • Adequate organ function. | • Prior exposure to trabectedin or dacarbazine.  • Myocardial infarct within 6 months before enrollment; NYHA Class II or greater heart failure, uncontrolled angina, severe uncontrolled ventricular arrhythmias, clinically significant pericardial disease, or electrocardiographic evidence of acute ischemic or active conduction system abnormalities.  • Uncontrolled intercurrent illness including, but not limited to, hypertension. |
| OVA-301  (NCT00113607) | • Histologically proven epithelial ovarian cancer, epithelial fallopian tube cancer, or primary peritoneal cancer.  • Patients with advanced ovarian cancer for whom initial platinum-based chemotherapy has failed.  • Prior treatment with only 1 chemotherapy regimen, including adjuvant therapy, which must be platinum-based and may include sequential maintenance therapy or second look debulking surgery (no gaps in administration of initial therapy may occur other than a delay of ≤2 months for treatment toxicity).  • ECOG performance status ≤2.  • Recurrence or progression after 6 full cycles of a complete 6 cycle initial treatment regimen or 6 months after the beginning (first dose) of the initial treatment line of platinum-based chemotherapy for ovarian cancer to include: patients with platinum-resistant disease (PFI <6 months) and patients with platinum-sensitive disease (PFI ≥6 months).  • Adequate organ function.  • LVEF must be within normal limits for the institution. | • Prior exposure to anthracyclines for ovarian cancer or to trabectedin.  • Myocardial infarct within 6 months before enrollment, NYHA Class II or greater heart failure, uncontrolled angina, severe uncontrolled ventricular arrhythmias, clinically significant pericardial disease, or electrocardiographic evidence of acute ischemic or active conduction system abnormalities.  • Acute deep vein thrombosis requiring intravenous or subcutaneous therapeutic anticoagulant therapy (chronic warfarin or prophylactic subcutaneous heparin allowed). |
| OVC-3006  (NCT01846611) | • Histologically proven advanced-relapsed epithelial ovarian, primary peritoneal, or fallopian tube cancer.  • ECOG performance status Grade 0 or 1.  • First-line treatment with a platinum-based regimen with no evidence of disease progression for ≥6 months after the last dose.  • Second-line treatment with a platinum-based regimen, with progression of disease after attaining a partial or complete response.  • Patients treated with a PLD-containing regimen as a second-line therapy were eligible if subsequent disease progression occurred ≥9 months from the first dose.  • Adequate organ function.  • LVEF within normal limits for the institution. | • Had >2 prior lines of systemic therapy.  • Prior treatment with doxorubicin or another anthracycline at a cumulative dose >300 mg/m^2^.  • Had a myocardial infarct within 6 months before enrollment, NYHA Class II or greater heart failure, uncontrolled angina, severe uncontrolled ventricular arrhythmias, clinically significant pericardial disease, or electrocardiographic evidence of acute ischemic or active conduction system abnormalities.  • Newly diagnosed deep vein thrombosis. |

ECOG, Eastern Cooperative Oncology Group; LVEF, left ventricular ejection fraction; NYHA, New York Heart Association; PFI, platinum-free interval; PLD, pegylated liposomal doxorubicin.

**Table S2. Key Cardiac Evaluations for Phase 3 Trials**

| **Protocol** | **Cardiac Evaluations** |
| --- | --- |
| SAR-3007 (NCT01343277) | • ECGs conducted at screening Phase and at the treatment termination visit. |
|  | • For LVEF assessments, MUGA scans were to be performed 30 days before randomization and as part of the end of treatment evaluations. Echocardiograms could be substituted for MUGA, if the latter was not available. The same methodology was to be used for both screening and end-of-treatment evaluations. |
| OVA-301 (NCT00113607) | • ECGs conducted at screening and at the treatment termination visit. |
|  | • For LVEF assessments, a MUGA scan or 2-d ECHO was to be performed within 6 weeks before enrollment and the LVEF was to be within normal range according to the institutional guidelines. Patients with a cardiac history or those who received a cumulative dose of anthracyclines that exceeded 360 mg/m^2^ were to have follow up MUGA scans or 2-d ECHOs after every 2 cycles of therapy. Each patient was to have a MUGA scan or 2-d ECHO at the treatment termination visit. The same methodology was to be used to assess LVEF in repeat evaluations. |
| OVC-3006^a^ (NCT01846611) | • ECGs conducted at screening and at the treatment termination visit. |
|  | • For LVEF assessments, a MUGA scan or 2-d ECHO was to be performed for all patients at screening, after an approximately 100 mg/m^2^ cumulative dose of planned anthracycline treatment with PLD (ie, after Cycle 3 for treatment Arm A [trabectedin+PLD combination therapy] and after Cycle 2 for treatment Arm B [PLD monotherapy]) and as part of the end-of-treatment termination visit. Patients who received a cumulative dose of anthracyclines that exceeded 300 mg/m^2^ (including previous and on-study treatments) or who had a clinically significant history of cardiomyopathy, were to have follow-up LVEF assessments after every 2 cycles of therapy. In addition, patients experiencing a significant LVEF decrease (defined as absolute decrease ≥15%, or less than lower limit of normal and absolute decrease ≥5%) not recovered to less than Grade 2 (or baseline) by the end of treatment were to have follow-up assessments of LVEF every 2 months until recovered or up to 6 months after discontinuation of study treatment (whichever occurred first). The same methodology was to be used to assess LVEF during the study. |

ECG, electrocardiogram; LVEF, left ventricular ejection fraction; MUGA, multigated acquisition scan; PLD, pegylated liposomal doxorubicin; 2‑d ECHO, 2‑dimensional echocardiogram.

^a^Evaluations based on protocol amendment 5. Per an Independent Review Committee recommendation, the protocol was amended (amendment 6; January 9, 2018) to prematurely terminate the study as futility for overall survival was met.

**Table S3. Number of Patients Receiving Trabectedin Monotherapy at 1.5 mg/m^2^ q3wk; 24 h (Trabectedin - Pooled Phase 2 and 3 Studies)**

| **Study No.** | **Patients Receiving Trabectedin**  **q3wk; 24 h (1.5 mg/m**^2^**)** | **Cancer Diagnosis** |
| --- | --- | --- |
| ET-B-005-98 | 126 (12.8) | STS (n=99), GIST (n=27) |
| ET-B-008-98 | 143 (14.6) | Breast (n=27), renal cancer (n=21), melanoma (n=12), STS (n=50), GIST (n=4), bone sarcoma (n=29) |
| ET-B-016-99 | 36 (3.7) | STS |
| ET-B-017-99 | 36 (3.7) | STS |
| ET-B-018-99 | 20 (2.0) | GIST |
| ET-B-019-99 | 25 (2.5) | Osteosarcoma |
| ET-B-025-02 (Cohort B1) | 5 (0.5) | Androgen-independent metastatic prostate cancer |
| ET-B-026-03 (Arm A) | 54 (5.5) | Ovarian |
| ET-B-028-06 | 29 (3.0) | Myxoid or round cell liposarcoma |
| ET743-STS-201 | 130 (13.2) | STS |
| ET743-SAR-3007 | 378 (38.5) | STS (leiomyosarcoma and liposarcoma) |
| Total^a^ | 982 |  |

GIST, gastrointestinal stromal tumors; q3wk; 24 h, once every 3 weeks as a 24-hour intravenous infusion; STS, soft tissue sarcoma.

Data are presented as No. (%) unless otherwise specified.

^a^All patients treated with trabectedin monotherapy q3wk; 24 h.

**Table S4. Demographics and Disease Characteristics for Patients Treated With Trabectedin 1.5 mg/m^2^ q3wk; 24 h (Trabectedin - Pooled Phase 2 and 3 Studies)**

|  | **Patients Treated With Trabectedin 1.5 mg/m^2^ q3wk; 24 h (N=982)** |
| --- | --- |
| Age, median (range), y | 54 (12, 81) |
| <18 | 11 (1.1) |
| 18 to <65 | 792 (80.7) |
| ≥65 | 179 (18.2) |
| Sex |  |
| Male | 377 (38.4) |
| Female | 605 (61.6) |
| Race |  |
| White | 497 (50.6) |
| Black | 57 (5.8) |
| Asian | 14 (1.4) |
| Other | 9 (0.9) |
| Unknown | 19 (1.9) |
| Not collected | 386 (39.3) |
| ECOG Performance Status | n=981 |
| 0 | 491 (50.1) |
| 1 | 488 (49.7) |
| 2 | 2 (0.2) |
| Abnormal Renal Function | n=980 |
| No | 901 (91.9) |
| Yes | 79 (8.1) |
| Abnormal Hepatic Function | n=981 |
| No | 687 (70.0) |
| Yes | 294 (30.0) |

ECOG, Eastern Cooperative Oncology Group; q3wk; 24 h, once every 3 weeks as a 24-hour intravenous infusion.

Data are presented as No. (%) unless otherwise specified.

**Table S5. Baseline Demographics for Treated Patients (Pooled Studies ET743-OVC-3006 and ET743-OVA-301)**

|  | **Trabectedin+PLD (N=619)** | **PLD (N=612)** |
| --- | --- | --- |
| Age, median (range), y | 58 (26, 83) | 59 (27, 91) |
| 18 to <65 | 431 (69.6) | 407 (66.5) |
| ≥65 | 188 (30.4) | 205 (33.5) |
| Race |  |  |
| White | 520 (84.0) | 502 (82.0) |
| Asian | 80 (12.9) | 93 (15.2) |
| Black or African American | 3 (0.5) | 4 (0.7) |
| Other | 8 (1.3) | 4 (0.7) |
| Unknown | 3 (0.5) | 1 (0.2) |
| Not Reported | 1 (0.2) | 3 (0.5) |
| American Indian/Alaska Native | 1 (0.2) | 1 (0.2) |
| Native Hawaiian or Other Pacific Islander | 1 (0.2) | 1 (0.2) |
| ECOG Performance Status |  |  |
| 0 | 375 (60.6) | 324 (52.9) |
| 1 | 235 (38.0) | 277 (45.3) |
| ≥2 | 9 (1.5) | 11 (1.8) |

ECOG, Eastern Cooperative Oncology Group; PLD, pegylated liposomal doxorubicin.

Data are presented as No. (%) unless otherwise specified.

**Table S6. Cardiac-Related Treatment-Emergent Adverse Events for Patients Treated With Trabectedin 1.5 mg/m^2^ q3wk; 24 h (Trabectedin - Pooled Phase 2 and 3 Studies)**

|  | **Patients Treated With Trabectedin 1.5 mg/m^2^ q3wk; 24 h (N=982)** |
| --- | --- |
| Total No. patients with cardiac-related TEAEs | 110 (11.2) |
| HLGT/SMQ  Preferred Term |  |
| Cardiac Arrhythmias (HLGT) | 65 (6.6) |
| Tachycardia | 30 (3.1) |
| Sinus tachycardia | 10 (1.0) |
| Atrial fibrillation | 9 (0.9) |
| Arrhythmia | 7 (0.7) |
| Atrial flutter | 5 (0.5) |
| Bradycardia | 3 (0.3) |
| Cardiac arrest | 3 (0.3) |
| Conduction disorder | 1 (0.1) |
| Extrasystoles | 1 (0.1) |
| Supraventricular extrasystoles | 1 (0.1) |
| Ventricular arrhythmia | 1 (0.1) |
| Ventricular extrasystoles | 1 (0.1) |
| Ventricular tachycardia | 1 (0.1) |
| Cardiac Failure (SMQ) | 32 (3.3) |
| Ejection fraction decreased | 13 (1.3) |
| Cardiac failure congestive | 10 (1.0) |
| Pulmonary oedema | 8 (0.8) |
| Cardiac failure | 7 (0.7) |
| Acute pulmonary oedema | 1 (0.1) |
| Cardiac failure acute | 1 (0.1) |
| Cardiogenic shock | 1 (0.1) |
| Left ventricular failure | 1 (0.1) |
| Right ventricular failure | 1 (0.1) |
| Cardiac and Vascular Investigations (Excluding Enzyme Tests) (HLGT) | 22 (2.2) |
| Ejection fraction decreased | 13 (1.3) |
| Cardiac murmur | 2 (0.2) |
| Electrocardiogram QT prolonged | 2 (0.2) |
| Heart rate increased | 2 (0.2) |
| Blood pressure decreased | 1 (0.1) |
| Blood pressure increased | 1 (0.1) |
| Heart rate abnormal | 1 (0.1) |
| Vascular resistance systemic | 1 (0.1) |
| Heart Failures (HLGT) | 20 (2.0) |
| Cardiac failure congestive | 10 (1.0) |
| Cardiac failure | 7 (0.7) |
| Cardiac failure acute | 1 (0.1) |
| Cardiogenic shock | 1 (0.1) |
| Left ventricular failure | 1 (0.1) |
| Right ventricular failure | 1 (0.1) |
| Cardiac Disorder Signs and Symptoms (HLGT) | 17 (1.7) |
| Palpitations | 15 (1.5) |
| Atrial thrombosis | 1 (0.1) |
| Cardiac disorder | 1 (0.1) |
| Cardiomyopathy (SMQ) | 15 (1.5) |
| Ejection fraction decreased | 13 (1.3) |
| Cardiomyopathy | 4 (0.4) |
| Myocardial Disorders (HLGT) | 9 (0.9) |
| Cardiomyopathy | 4 (0.4) |
| Cardiomegaly | 2 (0.2) |
| Diastolic dysfunction | 1 (0.1) |
| Left ventricular dysfunction | 1 (0.1) |
| Right ventricular dysfunction | 1 (0.1) |
| Pericardial Disorders (HLGT) | 7 (0.7) |
| Pericardial effusion | 6 (0.6) |
| Cardiac tamponade | 1 (0.1) |
| Coronary Artery Disorders (HLGT) | 4 (0.4) |
| Angina pectoris | 2 (0.2) |
| Acute myocardial infarction | 1 (0.1) |
| Myocardial infarction | 1 (0.1) |

HLGT, high-level group term; q3wk; 24 h, once every 3 weeks as a 24-hour intravenous infusion; SMQ, Standardized Medical Dictionary for Regulatory Activities (MedDRA) Query; TEAE, treatment-emergent adverse event.

Data are presented as No. (%). Adverse events reported any time from first treatment dose to within 30 days after last treatment dose are included. Adverse events were coded using MedDRA version 16.0.

**Table S7. Cardiac-Related Treatment-Emergent Grade 3 or 4 Adverse Events for Patients Treated With Trabectedin 1.5 mg/m^2^ q3wk; 24 h (Trabectedin - Pooled Phase 2 and 3 Studies)**

|  | **Patients Treated With Trabectedin 1.5 mg/m^2^ q3wk; 24 h (N=982)** | | |
| --- | --- | --- | --- |
|  | **Grade 3** | **Grade 4** | **Total Grade 3/4** |
| Total No. patients with Grade 3-4 TEAEs | 24 (2.4) | 13 (1.3) | 37 (3.8) |
| HLGT/SMQ |  |  |  |
| Preferred Term |  |  |  |
| Cardiac Failure (SMQ) | 17 (1.7) | 7 (0.7) | 24 (2.4) |
| Cardiac failure congestive | 9 (0.9) | 0 | 9 (0.9) |
| Ejection fraction decreased | 5 (0.5) | 3 (0.3) | 8 (0.8) |
| Cardiac failure | 3 (0.3) | 2 (0.2) | 5 (0.5) |
| Pulmonary oedema | 4 (0.4) | 1 (0.1) | 5 (0.5) |
| Cardiac failure acute | 0 | 1 (0.1) | 1 (0.1) |
| Cardiogenic shock | 0 | 1 (0.1) | 1 (0.1) |
| Left ventricular failure | 1 (0.1) | 0 | 1 (0.1) |
| Heart Failures (HLGT) | 12 (1.2) | 4 (0.4) | 16 (1.6) |
| Cardiac failure congestive | 9 (0.9) | 0 | 9 (0.9) |
| Cardiac failure | 3 (0.3) | 2 (0.2) | 5 (0.5) |
| Cardiac failure acute | 0 | 1 (0.1) | 1 (0.1) |
| Cardiogenic shock | 0 | 1 (0.1) | 1 (0.1) |
| Left ventricular failure | 1 (0.1) | 0 | 1 (0.1) |
| Cardiac Arrhythmias (HLGT) | 9 (0.9) | 3 (0.3) | 12 (1.2) |
| Atrial fibrillation | 4 (0.4) | 2 (0.2) | 6 (0.6) |
| Atrial flutter | 2 (0.2) | 0 | 2 (0.2) |
| Sinus tachycardia | 2 (0.2) | 0 | 2 (0.2) |
| Tachycardia | 1 (0.1) | 1 (0.1) | 2 (0.2) |
| Cardiomyopathy (SMQ) | 7 (0.7) | 3 (0.3) | 10 (1.0) |
| Ejection fraction decreased | 5 (0.5) | 3 (0.3) | 8 (0.8) |
| Cardiomyopathy | 3 (0.3) | 0 | 3 (0.3) |
| Cardiac and Vascular Investigations (Excluding Enzyme Tests) (HLGT) | 4 (0.4) | 4 (0.4) | 8 (0.8) |
| Ejection fraction decreased | 5 (0.5) | 3 (0.3) | 8 (0.8) |
| Electrocardiogram QT prolonged | 0 | 1 (0.1) | 1 (0.1) |
| Myocardial Disorders (HLGT) | 5 (0.5) | 0 | 5 (0.5) |
| Cardiomyopathy | 3 (0.3) | 0 | 3 (0.3) |
| Diastolic dysfunction | 1 (0.1) | 0 | 1 (0.1) |
| Left ventricular dysfunction | 1 (0.1) | 0 | 1 (0.1) |
| Cardiac Disorder Signs and Symptoms (HLGT) | 2 (0.2) | 0 | 2 (0.2) |
| Atrial thrombosis | 1 (0.1) | 0 | 1 (0.1) |
| Cardiac disorder | 1 (0.1) | 0 | 1 (0.1) |
| Coronary Artery Disorders (HLGT) | 0 | 2 (0.2) | 2 (0.2) |
| Acute myocardial infarction | 0 | 1 (0.1) | 1 (0.1) |
| Myocardial infarction | 0 | 1 (0.1) | 1 (0.1) |
| Pericardial Disorders (HLGT) | 1 (0.1) | 0 | 1 (0.1) |
| Cardiac tamponade | 1 (0.1) | 0 | 1 (0.1) |

HLGT, high-level group term; q3wk; 24 h, once every 3 weeks as a 24-hour intravenous infusion; SMQ, Standardized Medical Dictionary for Regulatory Activities (MedDRA) Query; TEAE, treatment-emergent adverse event.

Data are presented as No. (%). Adverse events reported any time from first treatment dose to within 30 days after last treatment dose are included. Adverse events were coded using MedDRA version 16.0.

**Table S8. Cardiac-Related Serious Treatment-Emergent Adverse Events for Patients Treated With Trabectedin 1.5 mg/m^2^ q3wk; 24 h (Trabectedin - Pooled Phase 2 and 3 Studies)**

|  | **Patients Treated With Trabectedin 1.5 mg/m^2^ q3wk; 24 h (N=982)** |
| --- | --- |
| Total No. patients with serious cardiac-related TEAEs | 36 (3.7) |
| HLGT/SMQ |  |
| Preferred Term |  |
| Cardiac Failure (SMQ) | 19 (1.9) |
| Cardiac failure congestive | 8 (0.8) |
| Pulmonary oedema | 6 (0.6) |
| Cardiac failure | 5 (0.5) |
| Ejection fraction decreased | 5 (0.5) |
| Cardiac failure acute | 1 (0.1) |
| Cardiac Arrhythmias (HLGT) | 16 (1.6) |
| Atrial fibrillation | 5 (0.5) |
| Cardiac arrest | 3 (0.3) |
| Arrhythmia | 2 (0.2) |
| Atrial flutter | 2 (0.2) |
| Tachycardia | 2 (0.2) |
| Conduction disorder | 1 (0.1) |
| Sinus tachycardia | 1 (0.1) |
| Ventricular tachycardia | 1 (0.1) |
| Heart Failures (HLGT) | 13 (1.3) |
| Cardiac failure congestive | 8 (0.8) |
| Cardiac failure | 5 (0.5) |
| Cardiac failure acute | 1 (0.1) |
| Cardiomyopathy (SMQ) | 7 (0.7) |
| Ejection fraction decreased | 5 (0.5) |
| Cardiomyopathy | 3 (0.3) |
| Cardiac and Vascular Investigations (Excluding Enzyme Tests) (HLGT) | 5 (0.5) |
| Ejection fraction decreased | 5 (0.5) |
| Myocardial Disorders (HLGT) | 5 (0.5) |
| Cardiomyopathy | 3 (0.3) |
| Left ventricular dysfunction | 1 (0.1) |
| Right ventricular dysfunction | 1 (0.1) |
| Cardiac Disorder Signs and Symptoms (HLGT) | 2 (0.2) |
| Cardiac disorder | 1 (0.1) |
| Palpitations | 1 (0.1) |
| Coronary Artery Disorders (HLGT) | 2 (0.2) |
| Acute myocardial infarction | 1 (0.1) |
| Myocardial infarction | 1 (0.1) |
| Pericardial Disorders (HLGT) | 1 (0.1) |
| Cardiac tamponade | 1 (0.1) |

HLGT, high-level group term; q3wk; 24 h, once every 3 weeks as a 24-hour intravenous infusion; SMQ, Standardized Medical Dictionary for Regulatory Activities (MedDRA) Query; TEAE, treatment-emergent adverse event.

Data are presented as No. (%). Adverse events reported any time from first treatment dose to within 30 days after last treatment dose are included. Adverse events were coded using MedDRA version 16.0.

**Table S9. Cardiac-Related Treatment-Emergent Adverse Events Leading to Death for Patients Treated With Trabectedin 1.5 mg/m^2^ q3wk; 24 h (Trabectedin - Pooled Phase 2 and 3 Studies)**

|  | **Trabectedin 1.5 mg/m^2^ q3wk; 24 h (N=982)** |
| --- | --- |
| Total No. patients with cardiac-related TEAEs leading to death | 6 (0.6) |
| HLGT/SMQ |  |
| Preferred Term |  |
| Cardiac Arrhythmias (HLGT) | 4 (0.4) |
| Cardiac arrest | 3 (0.3) |
| Tachycardia | 1 (0.1) |
| Cardiac Failure (SMQ) | 1 (0.1) |
| Cardiac failure | 1 (0.1) |
| Coronary Artery Disorders (HLGT) | 1 (0.1) |
| Acute myocardial infarction | 1 (0.1) |
| Heart Failures (HLGT) | 1 (0.1) |
| Cardiac failure | 1 (0.1) |

HLGT, high-level group term; q3wk; 24 h, once every 3 weeks as a 24-hour intravenous infusion; SMQ, Standardized Medical Dictionary for Regulatory Activities (MedDRA) Query; TEAE, treatment-emergent adverse event.

Data are presented as No. (%). Adverse events reported any time from first treatment dose to within 30 days after last treatment dose are included. Cardiac-related TEAEs leading to death is defined as cardiac-related TEAEs with an outcome of death. Adverse events were coded using MedDRA version 16.0.

**Table S10. Cardiac-Related Treatment-Emergent Adverse Events for Treated Patients (Pooled Studies ET743-OVC-3006 and ET743-OVA-301)**

|  | **Trabectedin+PLD (N=619)** | **PLD (N=612)** |
| --- | --- | --- |
| Total No. patients with cardiac-related TEAEs | 78 (12.6) | 34 (5.6) |
| HLGT/SMQ |  |  |
| Preferred Term |  |  |
| Cardiac Failure (SMQ) | 31 (5.0) | 15 (2.5) |
| Ejection fraction decreased | 24 (3.9) | 13 (2.1) |
| Cardiac failure congestive | 4 (0.6) | 1 (0.2) |
| Pulmonary oedema | 3 (0.5) | 1 (0.2) |
| Cardiac failure | 2 (0.3) | 0 |
| Cardiopulmonary failure | 1 (0.2) | 0 |
| Cardiac and Vascular Investigations (Excluding Enzyme Tests) (HLGT) | 27 (4.4) | 13 (2.1) |
| Ejection fraction decreased | 24 (3.9) | 13 (2.1) |
| Electrocardiogram change | 1 (0.2) | 0 |
| Heart rate increased | 1 (0.2) | 0 |
| Heart rate irregular | 1 (0.2) | 0 |
| Cardiomyopathy (SMQ) | 26 (4.2) | 13 (2.1) |
| Ejection fraction decreased | 24 (3.9) | 13 (2.1) |
| Cardiomyopathy | 2 (0.3) | 0 |
| Cardiac Arrhythmias (HLGT) | 21 (3.4) | 12 (2.0) |
| Tachycardia | 14 (2.3) | 7 (1.1) |
| Sinus tachycardia | 3 (0.5) | 0 |
| Atrial fibrillation | 1 (0.2) | 2 (0.3) |
| Bundle branch block right | 1 (0.2) | 0 |
| Cardiac arrest | 1 (0.2) | 0 |
| Pulseless electrical activity | 1 (0.2) | 0 |
| Ventricular tachycardia | 1 (0.2) | 0 |
| Bradycardia | 0 | 1 (0.2) |
| Bundle branch block | 0 | 1 (0.2) |
| Cardiac flutter | 0 | 1 (0.2) |
| Sinus arrhythmia | 0 | 1 (0.2) |
| Cardiac Disorder Signs and Symptoms (HLGT) | 21 (3.4) | 6 (1.0) |
| Palpitations | 20 (3.2) | 6 (1.0) |
| Cardiac disorder | 1 (0.2) | 0 |
| Myocardial Disorders (HLGT) | 9 (1.5) | 2 (0.3) |
| Left ventricular dysfunction | 4 (0.6) | 0 |
| Cardiomyopathy | 2 (0.3) | 0 |
| Diastolic dysfunction | 2 (0.3) | 1 (0.2) |
| Ventricular dysfunction | 1 (0.2) | 0 |
| Left ventricular hypertrophy | 0 | 1 (0.2) |
| Heart Failures (HLGT) | 7 (1.1) | 1 (0.2) |
| Cardiac failure congestive | 4 (0.6) | 1 (0.2) |
| Cardiac failure | 2 (0.3) | 0 |
| Cardiopulmonary failure | 1 (0.2) | 0 |
| Coronary Artery Disorders (HLGT) | 3 (0.5) | 2 (0.3) |
| Angina pectoris | 2 (0.3) | 1 (0.2) |
| Myocardial ischaemia | 1 (0.2) | 1 (0.2) |
| Pericardial Disorders (HLGT) | 2 (0.3) | 0 |
| Pericardial effusion | 2 (0.3) | 0 |

HLGT, high-level group term; SMQ, Standardized Medical Dictionary for Regulatory Activities (MedDRA) Query; PLD, pegylated liposomal doxorubicin; TEAE, treatment-emergent adverse event.

Data are presented as No. (%). Left ventricular ejection fraction significant decrease is defined as absolute decrease ≥15%, or less than lower limit of normal, and absolute decrease ≥5%. Adverse events reported any time from first treatment dose to within 30 days after last treatment dose are included. Adverse events were coded using MedDRA version 19.0.

**Table S11. Cardiac-Related Treatment-Emergent Grade 3 or 4 Adverse Events for Treated Patients (Pooled Studies ET743-OVC-3006 and ET743-OVA-301)**

|  | **Trabectedin+PLD (N=619)** | | | **PLD (N=612)** | | |
| --- | --- | --- | --- | --- | --- | --- |
|  | **Total Grade 3/4** | **Grade 3** | **Grade 4** | **Total Grade 3/4** | **Grade 3** | **Grade 4** |
| Total No. patients with Grade 3-4 TEAEs | 14 (2.3) | 12 (1.9) | 2 (0.3) | 4 (0.7) | 3 (0.5) | 1 (0.2) |
| HLGT/SMQ |  |  |  |  |  |  |
| Preferred Term |  |  |  |  |  |  |
| Cardiac Failure (SMQ) | 8 (1.3) | 7 (1.1) | 1 (0.2) | 2 (0.3) | 1 (0.2) | 1 (0.2) |
| Ejection fraction decreased | 5 (0.8) | 5 (0.8) | 0 | 1 (0.2) | 1 (0.2) | 0 |
| Cardiac failure congestive | 3 (0.5) | 2 (0.3) | 1 (0.2) | 1 (0.2) | 0 | 1 (0.2) |
| Cardiac failure | 1 (0.2) | 1 (0.2) | 0 | 0 | 0 | 0 |
| Pulmonary oedema | 1 (0.2) | 1 (0.2) | 0 | 0 | 0 | 0 |
| Cardiac and Vascular Investigations (Excluding Enzyme Tests) (HLGT) | 5 (0.8) | 5 (0.8) | 0 | 1 (0.2) | 1 (0.2) | 0 |
| Ejection fraction decreased | 5 (0.8) | 5 (0.8) | 0 | 1 (0.2) | 1 (0.2) | 0 |
| Cardiomyopathy (SMQ) | 5 (0.8) | 5 (0.8) | 0 | 1 (0.2) | 1 (0.2) | 0 |
| Ejection fraction decreased | 5 (0.8) | 5 (0.8) | 0 | 1 (0.2) | 1 (0.2) | 0 |
| Heart Failures (HLGT) | 4 (0.6) | 3 (0.5) | 1 (0.2) | 1 (0.2) | 0 | 1 (0.2) |
| Cardiac failure congestive | 3 (0.5) | 2 (0.3) | 1 (0.2) | 1 (0.2) | 0 | 1 (0.2) |
| Cardiac failure | 1 (0.2) | 1 (0.2) | 0 | 0 | 0 | 0 |
| Cardiac Arrhythmias (HLGT) | 3 (0.5) | 2 (0.3) | 1 (0.2) | 1 (0.2) | 1 (0.2) | 0 |
| Atrial fibrillation | 1 (0.2) | 1 (0.2) | 0 | 1 (0.2) | 1 (0.2) | 0 |
| Cardiac arrest | 1 (0.2) | 0 | 1 (0.2) | 0 | 0 | 0 |
| Pulseless electrical activity | 1 (0.2) | 0 | 1 (0.2) | 0 | 0 | 0 |
| Tachycardia | 1 (0.2) | 1 (0.2) | 0 | 0 | 0 | 0 |
| Cardiac Disorder Signs and Symptoms (HLGT) | 2 (0.3) | 2 (0.3) | 0 | 0 | 0 | 0 |
| Cardiac disorder | 1 (0.2) | 1 (0.2) | 0 | 0 | 0 | 0 |
| Palpitations | 1 (0.2) | 1 (0.2) | 0 | 0 | 0 | 0 |
| Coronary Artery Disorders (HLGT) | 1 (0.2) | 1 (0.2) | 0 | 0 | 0 | 0 |
| Angina pectoris | 1 (0.2) | 1 (0.2) | 0 | 0 | 0 | 0 |
| Myocardial Disorders (HLGT) | 1 (0.2) | 1 (0.2) | 0 | 1 (0.2) | 1 (0.2) | 0 |
| Left ventricular dysfunction | 1 (0.2) | 1 (0.2) | 0 | 0 | 0 | 0 |
| Left ventricular hypertrophy | 0 | 0 | 0 | 1 (0.2) | 1 (0.2) | 0 |

HLGT, high-level group term; PLD, pegylated liposomal doxorubicin; SMQ, Standardized Medical Dictionary for Regulatory Activities (MedDRA) Query; TEAE, treatment-emergent adverse event.

Data are presented as No. (%). Left ventricular ejection fraction significant decrease is defined as absolute decrease ≥15%, or less than lower limit of normal and absolute decrease ≥5%. Adverse events reported any time from first treatment dose to within 30 days after last treatment dose are included. Adverse events were coded using MedDRA version 19.0.

**Table S12. Cardiac-Related Serious Treatment-Emergent Adverse Events for Treated Patients (Pooled Studies ET743-OVC-3006 and ET743-OVA-301)**

|  | **Trabectedin+PLD (N=619)** | **PLD (N=612)** |
| --- | --- | --- |
| Total No. patients with serious cardiac-related TEAEs | 11 (1.8) | 3 (0.5) |
| HLGT/SMQ |  |  |
| Preferred Term |  |  |
| Cardiac Failure (SMQ) | 6 (1.0) | 2 (0.3) |
| Cardiac failure congestive | 3 (0.5) | 1 (0.2) |
| Ejection fraction decreased | 2 (0.3) | 1 (0.2) |
| Cardiopulmonary failure | 1 (0.2) | 0 |
| Cardiac Arrhythmias (HLGT) | 4 (0.6) | 1 (0.2) |
| Atrial fibrillation | 1 (0.2) | 1 (0.2) |
| Cardiac arrest | 1 (0.2) | 0 |
| Pulseless electrical activity | 1 (0.2) | 0 |
| Tachycardia | 1 (0.2) | 0 |
| Ventricular tachycardia | 1 (0.2) | 0 |
| Heart Failures (HLGT) | 4 (0.6) | 1 (0.2) |
| Cardiac failure congestive | 3 (0.5) | 1 (0.2) |
| Cardiopulmonary failure | 1 (0.2) | 0 |
| Cardiac and Vascular Investigations (Excluding Enzyme Tests) (HLGT) | 3 (0.5) | 1 (0.2) |
| Ejection fraction decreased | 2 (0.3) | 1 (0.2) |
| Electrocardiogram change | 1 (0.2) | 0 |
| Cardiomyopathy (SMQ) | 2 (0.3) | 1 (0.2) |
| Ejection fraction decreased | 2 (0.3) | 1 (0.2) |
| Cardiac Disorder Signs and Symptoms (HLGT) | 1 (0.2) | 0 |
| Palpitations | 1 (0.2) | 0 |

HLGT, high-level group term; PLD, pegylated liposomal doxorubicin; SMQ, Standardized Medical Dictionary for Regulatory Activities (MedDRA) Query; TEAE, treatment-emergent adverse event.

Data are presented as No. (%). Left ventricular ejection fraction significant decrease is defined as absolute decrease ≥15%, or less than lower limit of normal and absolute decrease ≥5%. Adverse events reported any time from first treatment dose to within 30 days after last treatment dose are included. Adverse events were coded using MedDRA version 19.0.
